# Infection Load and Prevalence of Novel Viruses Identified from the Bank Vole Do Not Associate with Exposure to Environmental Radioactivity

**DOI:** 10.3390/v12010044

**Published:** 2019-12-30

**Authors:** Jenni Kesäniemi, Anton Lavrinienko, Eugene Tukalenko, Tapio Mappes, Phillip C. Watts, Jaana Jurvansuu

**Affiliations:** 1 Finland Ecology and Genetics Research Unit, University of Oulu, 90014 Oulu, Finland; jenni.e.kesaniemi@jyu.fi; 2 Department of Biological and Environmental Science, University of Jyväskylä, 40014 Jyväskylä, Finland; anton.lavrinienko@oulu.fi (A.L.); tapio.mappes@jyu.fi (T.M.); phillip.c.watts@jyu.fi (P.C.W.); 3 National Research Center for Radiation Medicine of the National Academy of Medical Science, 02000 Kyiv, Ukraine; etukalenko@gmail.com

**Keywords:** plasma viromes, bank vole, adeno-associated virus, mosavirus, arterivirus, environmental radiation, next-generation sequencing

## Abstract

Bank voles (*Myodes glareolus*) are host to many zoonotic viruses. As bank voles inhabiting areas contaminated by radionuclides show signs of immunosuppression, resistance to apoptosis, and elevated DNA repair activity, we predicted an association between virome composition and exposure to radionuclides. To test this hypothesis, we studied the bank vole virome in samples of plasma derived from animals inhabiting areas of Ukraine (contaminated areas surrounding the former nuclear power plant at Chernobyl, and uncontaminated areas close to Kyiv) that differed in level of environmental radiation contamination. We discovered four strains of hepacivirus and four new virus sequences: two adeno-associated viruses, an arterivirus, and a mosavirus. However, viral prevalence and viral load, and the ability to cause a systemic infection, was not dependent on the level of environmental radiation.

## 1. Introduction

Metagenomic analyses of rodent fecal and pharyngeal viromes indicate that rodents host a diverse community of DNA and RNA viruses, with viral diversity apparently depending on host taxonomy more than geographical location, although rodent viruses can often shift between hosts [1,2]. The bank vole (*Myodes glareolus*), a small rodent that is common in woodland habitats throughout much of Europe and northwest Asia, is an zoonotic hyper-reservoir [3]. Bank voles host disease-causing viruses such as Puumala hantavirus [4], flavivirus tick borne encephalitis virus [5], orthonairovirus Crimean–Congo hemorrhagic fever virus [6], cowpox virus [7], and picornavirus Ljungan virus [8]; moreover, bank voles harbor paramyxoviruses [9,10], alphacoronaviruses [11], polyomaviruses [12], herpesviruses [13,14], anelloviruses [15], and hepaciviruses [16]. As bank voles host a diverse and important viral community, this species is an ideal model in which to study the potential effects of environmental factors on virus community and population dynamics. For example, anthropogenically driven environmental changes have been shown to affect Puumala hantaviral transmission in rodents including bank vole [17,18].

The effects of environmental radiation on host–viral dynamics in natural systems are unknown. While high doses (in excess of several kilograys, kGy) of ionizing radiation inactivate viruses [19,20], exposure to lower doses (2.5–25 Gy) can activate replication in some viruses, such as cytomegalovirus [21], endogenous retroviruses [22], human immunodeficiency virus [23], hepatitis B virus [24,25], Epstein–Barr virus [26,27], reovirus [28], herpes simplex virus [29], parvoviruses [30,31], and poliovirus [32]. Virus activation by irradiation may be facilitated by radiation-mediated immunosuppression that makes the host more susceptible to virus infection and replication [33,34,35], or by radiation-induced activation of the DNA repair proteins that some viruses utilize in their replication [36,37]. Wild bank voles exposed to chronic low-dose (up to 20 µGy/h) environmental radiation show activated transcription of DNA repair proteins [38] and inhibited apoptosis [39], and show signs of immunosuppression [40]. As these processes may support virus replication, we predicted an elevated viral load in bank voles inhabiting areas contaminated by radionuclides compared with uncontaminated areas.

To test our prediction, we isolated and characterized the virome from plasma samples from bank voles inhabiting (1) the Chernobyl exclusion zone (CEZ) and (2) from areas close to Kyiv, Ukraine, where the habitat is not contaminated by radionuclides. The CEZ is an area surrounding the former Chernobyl nuclear power plant that presents a mosaic of areas contaminated by radionuclides [41]. We identified four novel virus sequences in bank voles: Myodes glareolus adeno-associated viruses 1 and 2, Myodes glareolus arterivirus 1, and Myodes glareolus mosavirus 1. We also identified four new hepacivirus strains. Counter to our expectations, we found no evidence of an association between exposure to environmental radiation and either viral load (of plasma, and also derived from liver and spleen transcriptomes) or prevalence in bank voles.

## 2. Materials and Methods

### 2.1. Animals

**Figure 1:**
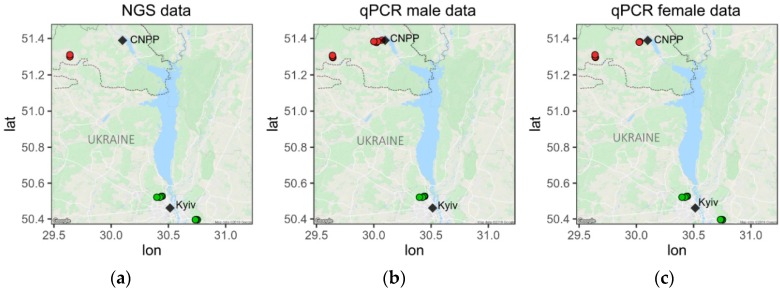
Sample collection sites for the (**a**) next-generation sequencing and quantitative PCR studies of (**b**) males and (**c**) females. Map of the study areas with bank vole trapping locations shown by points. The control areas (green) near Kyiv, and areas contaminated with radionuclides (red) within the Chernobyl exclusion zone in Ukraine are shown. Dashed line represent the border around the exclusion zone in Ukraine (area of ~2050 km^2^). Figure was created using ggmap v.3.0 package in R. CNPP shows the location of the Chernobyl nuclear power plant.

Bank voles were collected in July and October 2016. Bank voles were caught at 20 trapping locations within northern Ukraine (Tables S1–S3, Figure 1), using Ugglan Special2 live traps (Grahnab, Sweden) as previously described [42]. Briefly, at each location, 16 traps were placed in a 4 × 4 grid, with an intertrap distance of 20 m. The trapping period was up to three consecutive nights in each location. Traps were initially set in the late afternoon and animals were collected early each following morning. Captured animals were taken to a laboratory, where body mass and sex were recorded for all individuals. Blood sample was taken from the retro-orbital sinus with EDTA-treated capillaries (Hirschmann Laborgeräte, Germany) into EDTA-containing tubes (MiniCollector, Greiner Bio-One). Animals were humanely euthanized by cervical dislocation. Collected blood samples were centrifuged at 2000 *g* for 10 min, after which the supernatant was pipetted into sterile 1.5 mL tubes and frozen at −20 °C until further processing. Environmental radiation levels at each trapping location were measured at 1 cm above the ground using a Geiger–Mueller dosimeter (Gamma-Scout, Germany). In addition, every individual was subjected to γ-spectrometry (SAM 940, Berkeley Nucleonics) to estimate whole-body radionuclide (^137^Cs) burden and internal radiation exposure (see the Appendix A for details on estimates of the total absorbed radiation dose rates).

### 2.2. Virus Identification

Virus particles were isolated from pooled plasma samples. A total 100 µL of plasma was pooled from bank voles collected from the Kyiv control area (east: *N* = 9 and west: *N* = 8) or CEZ area (west: *N* = 10) (Figure 1, Table S1). Samples were centrifuged at 10,000 *g* for 10 min and supernatant was treated with universal nuclease (125 U/mL, Thermofisher, Waltham, MA, USA) at room temperature for 10 min. Samples were then filtered (0.45 µm) and centrifuged with a 38% sucrose cushion at 175,000 *g* for 3 h. Virus DNA and RNA was isolated from the pellet using a GeneJET Viral DNA/RNA Purification Kit (ThermoFisher). Double-stranded cDNA was synthesized with Maxima H Minus Double-Stranded cDNA Synthesis Kit (ThermoFisher); the samples were concentrated with PureLink^TM^ PCR Micro PCR kit, and sent for sequencing (100 bp paired-end reads on an Illumina HiSeq4000) to BGI Tech Solutions (Hong Kong, China).

Raw reads were deposited on the National Center for Biotechnolgy Information’s (NCBI) Sequence Read Archive (SRA) (bioproject ID: PRJNA557363). Servers and software for bioinformatic processing and analysis were provided by The Finnish IT center for Science (www.csc.fi). Adaptor sequences and low quality reads were removed using Trimmomatic [43]. Trinity v2.3.2 [44] was used to build contigs, CAP3 [45] was used to collapse overlapping contigs, Blastx v2.3.0 [46] and NCBI viral refseq (downloaded 01.05.2019) were used to identify contigs with virus sequences in them, and NOVOplasty [47] was used to lengthen the viral contigs. Read alignments on virus sequences were inspected with IGV [48], and only apparently complete or nearly complete (MgAAV2) virus sequences were used in further analysis. ORFfinder (www.ncbi.nlm.nih.gov/orffinder), Blastp (nr), and Hhpred [49] were used to identify coding regions and hypothetical protein function. mFold [50] was used to predict secondary RNA and DNA structures and TMHMM Server v. 2.0 (www.cbs.dtu.dk/services/TMHMM) was used to predict transmembrane domains. Remapping and mapping of reads from the transcriptome data to virus sequences was done with BWA [51].

Phylogenetic clustering of new viral genomes was achieved by aligning the five most similar polymerase sequences as identified by Blastp (nr) with Muscle [52] using neighbour-joining, Poisson correction, and 500 replicate bootstraps, as implemented in MEGA7 [53]. All positions containing gaps and missing data were eliminated.

### 2.3. qPCR

Virus DNA and RNA was isolated with GeneJET Viral DNA/RNA Purification Kit (ThermoFisher) from 100 µl of plasma (Kyiv east: *N*_females_ = 9, *N*_males_ = 10; Kyiv west: *N*_females_ = 10; CEZ east: *N*_females_ = 3, *N*_males_ = 9; and CEZ west: *N*_females_ = 17, *N*_males_ = 10; Tables S2 and S3, and Figure 1b,c). Prevalences and load of DNA viruses were quantified by qPCR on RNAse A- (ThermoFisher) treated and cleaned (GeneJET PCR purification Kit, ThermoFisher) DNA samples. To analyze RNA viruses, cDNA was made with RevertAid First Strand cDNA Synthesis Kit and random primers (ThermoFisher). Virus cDNA and RNAse-A-treated virus preparations were analyzed by qPCR with virus-specific primers (Table S6) using EvaGreen (Solis BioDyne), as recommended by the manufacturer. We used a purified (GeneJet gel Extraction kit, ThermoFisher) and quantified Phusion-amplified (ThermoFisher) PCR products as qPCR standards. Only results with more than 100 virus molecules per 2 µL sample were analyzed. 

### 2.4. Statistical Analysis

All statistical analyses were done in R v3.5.0 (www.r-project.org).

## 3. Results

### 3.1. Virus Identification from the Next-Generation Sequencing Data

**Figure 2:**
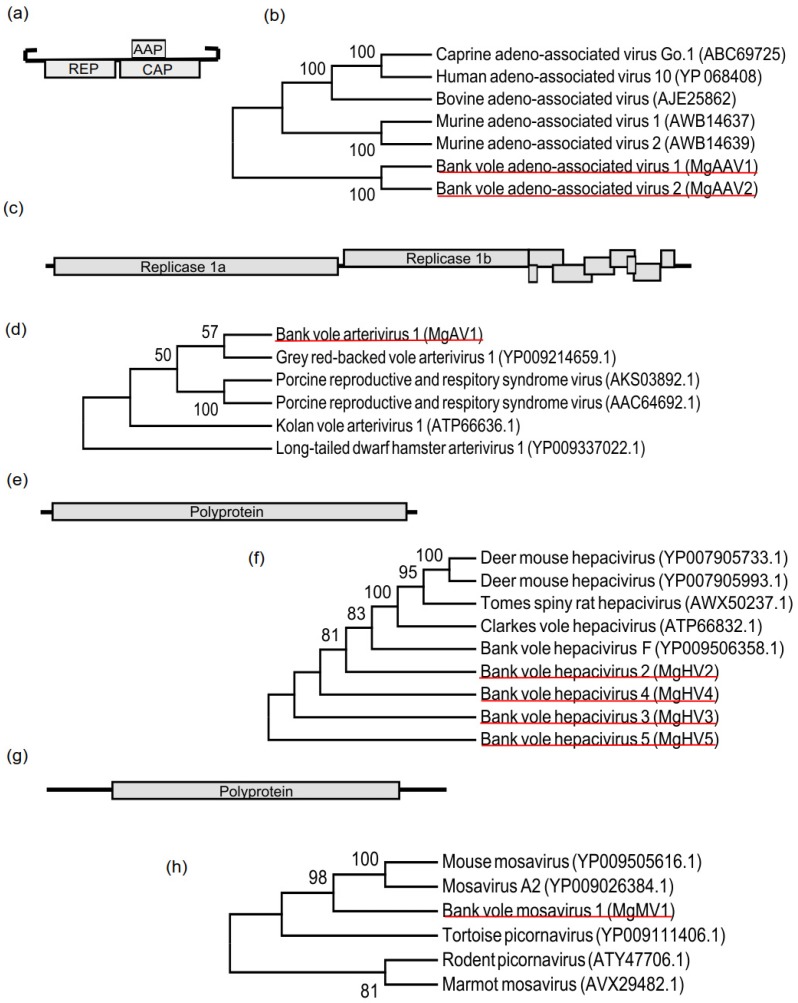
Genome organization and phylogenetic clustering of identified bank vole viruses: (**a**) and (**b**) MgAAV1 and MgAAV2, (**c**) and (**d**) MgAV1, (**e**) and (**f**) MgHV2–5, and (**g**) and (**h**) MgMV1. Virus genomes are drawn to scale: the largest virus, MgAV1, is 15.4 kb and the smallest, MgAAV, is 4.5 kb. Lines denote virus genomic sequence and boxes denote open reading frames. Different reading frames are presented by boxes aligning above, below, or on the line. Phylogenetic tree is a bootstrap consensus tree and the percentage of replicate trees (*N* = 500) in which taxa clustered together are shown next to the branches (only percentages over 50 are shown).

We isolated virus particles for sequencing from plasma of female bank voles collected from two areas near Kyiv (Kyiv N_west_ = 9, Kyiv N_east_ = 8), where there is no elevated level of radiation, and from a site within the Chernobyl exclusion zone (CEZ N_west_ = 10), where soil was contaminated with radionuclides (Figure 1A, Table S1). After next-generation sequencing on an Illumina platform, we assembled contigs from the pooled sequencing data using Trinity. Potential virus sequences were identified by their similarity to known viral proteins in NCBI GenBank database. We identified eight complete or nearly complete virus sequences (Table 1), which were classified according to their genomic organization and phylogenetic relationship to known mammalian viruses (Figure 2a–h): Myodes glareolus adeno-associated virus 1 (MgAAV1, Genbank ID = MN242366), Myodes glareolus adeno-associated virus 2 (MgAAV2, Genbank ID = MN242367), Myodes glareolus arterivirus 1 (MgAV1, Genbank ID = MN242368), Myodes glareolus hepacivirus 2–5 (MgHV2–5, Genbank IDs = MN242369, MN242370, MN242371, and MN242372 respectively), and Myodes glareolus mosavirus 1 (MgMV1, Genbank ID = MN242373).

### 3.2. Adeno-Associated Viruses

*Parvoviridae* are linear, single-stranded DNA viruses with a genome of 4–6 kb that codes for structural proteins (capsid) and replication protein (REP). Parvoviruses are non-enveloped viruses with two terminal hairpin structures, which prime replication [39]. Parvoviruses infect vertebrate (*Parvovirinae*) and invertebrate (*Densovirinae*) hosts [40]. The subfamily *Parvovirinae* is divided into eight genera based on the proteins they transcribe: *Amdoparvovirus*, *Aveparvovirus*, *Bocaparvovirus*, *Dependoparvovirus*, *Erythroparvovirus*, *Copiparvovirus*, *Protoparvovirus*, and *Tetraparvovirus* [54]. Dependoparvoviruses encode an additional small assembly-activating protein (AAP) from an alternative reading frame overlapping the structural protein. We found two parvovirus sequences with replication protein similarities to adeno-associated viruses of *Dependoparvovirus* genera; Myodes glareolus adeno-associated virus 1 (MgAAV1) and 2 (MgAAV2) had 4927 nt and 4517 nt long genomes, respectively (Figure 2a). MgAAV1 and 2 had 72% of identity between their replication proteins. According to International Committee on Taxonomy of Viruses (ICTV), the species demarcation criteria for dependoparvoviruses is less than 85% sequence identity on REP proteins, and thus MgAAV1 and 2 represent separate species. In the phylogenetic analysis, MgAAV1 and 2 sequences clustered with murine adeno-associated virus 1 and 2 (identified from fecal samples of New York city house mice [55]) and then with bovine, caprine, and human adeno-associated viruses [56] (Figure 2b). MgAAV1 had three open reading frames (ORFs) coding for 113 aa, 593 aa, and 716 aa long proteins. The smallest ORF of MgAAV1 encoded a protein similar to the AAP protein of human adeno-associated virus (ASW20948.1, 72% query cover, 61% identity), while the 593 aa long protein was similar to the REP of human adeno-associated virus 1 (AWB14637.1, 99% cover, 54.4% identity); MgAAV1′s largest protein was similar to murine adeno-associated virus 2 capsid (AWB14640.1, 100% cover, 63.7% identity). Similarly, MgAAV2 had three ORFs: 137 aa, 556 aa, and 729 aa in length. They were coding proteins similar to AAP of corn snake parvovirus (AKM49968.1, 97% cover, 54.3% identity), murine adeno-associated virus 1 REP (AWB14637.1, 87% cover, 62% identity), and capsid protein of bovine adeno-associated virus (AJE25863.1, 100% cover, 73% identity). Both viruses had inverted repeats in their genome: MgAAV1 at positions 1–330 nt and 4597–4927 nt and MgAAV2 at position 1–60. Since the 3′ hairpin structure of MgAAV2 was absent, the genome sequence was only partially reconstructed.

### 3.3. Arterivirus

*Arteriviruses* are enveloped, positive-strand RNA viruses in the order of *Nidovirales*. The *Arteriviridae* infect mammals, including Grey red-backed voles [1]. *Arteriviruses* infect macrophages and the infection can be asymptotic, persistent, or disease-associated [57]. *Arterivirus* genome is polycistronic, poly-adenylated, and codes for 11 proteins. We found nine ORFs from MgAV1, with the largest proteins (2491 and 1410 aa ORFs) similar to replicase polyprotein 1a and 1b of the Grey red-backed vole arterivirus 1 (YP009214659.1, 99% cover, 64% and 81.6% identity, respectively) (Figure 2c). MgAV1 clustered in phylogenetic analysis with the arterivirus identified from Grey red-backed vole [1] (Figure 2d) and is thus, according to ICTV nomenclature guidelines, best described as an unassigned species within the *Arteriviridae* family. The remaining ORFs of MgAV1 were (in order from the 5′ end): envelope protein E (70 aa, 100% cover, 55.7% identity with Clarke’s vole YP009551704.1), envelope glycoprotein 2 (232 aa, 74% coverage, 51.5% identity with Clarke’s vole YP009551705.1), envelope glycoprotein 3 (265 aa, 76% coverage, 39.5% identity with Clarke’s vole YP009551706.1), envelope glycoprotein 4 (183 aa, 98% coverage, 46.7% identity with Clarke’s vole YP009551707.1), envelope glycoprotein 5 (221 aa, 86% cover, 84.9% identity with Grey red-backed vole YP009214664.1), envelope protein 5a (45 aa, 100% cover, 66.7% identity with a long-tailed dwarf hamster YP009337028.1), membrane protein (174 aa, 100% cover, 91.4% identity with Grey red-backed vole YP009214665.1), and nucleocapsid protein (122 aa, 100% cover, 89.4% identity with Grey red-backed vole YP009214666.1). Surprisingly, our draft genome MgAV1 sequence did not contain the nonstructural protein 2 transframe fusion product (nsp2TF), which most of the identified arteriviruses had. The MgAV1 sequence had a 19 nt long poly-A tail. ICTV species demarcation criteria states that each arterivirus species is host-specific.

### 3.4. Hepaciviruses

Hepaciviruses are positive-strand RNA viruses that belong to the *Flaviviridae* family. Hepaciviruses infect a range of mammals, including bank voles [16]. From the three already identified bank vole hepaciviruses, one species has been suggested to cause liver inflammation [16]. We found four new hepacivirus sequences, which had genome sizes of 8900 (MgHV2), 8838 (MgHV3), 8829 (MgHV4), and 8828 (MgHV5) nt (Figure 2e). The bank vole hepaciviruses were most similar in nucleotide sequence to each other (100% cover, 85.5–89.9% identity) and then to bank vole hepacivirus F (100% coverage, 79.4–79.7% identity, NC_038427.1, isolated from a bank vole in the Netherlands [14]). In phylogenetic analysis, the Ukrainian bank vole hepacivirus strains clustered with hepacivirus F (YP009506358.1) and then to other rodent hepaciviruses (Figure 2f). Hepacivirus genome encodes for a multifunctional polyprotein that is comprised of four structural (C, E1, E2, p7/p13) and seven non-structural proteins (NS2, NS3, NS3, NS4a, NS4b, NS5a, and NS5b), which are cleaved by viral and host proteases [45]. Using Blastp, we identified conserved domains in each of the novel MgHV for structural nucleocapsid core protein (C) and envelope protein 1 (E1) and non-structural conserved domains for protease/helicase/NTPase (NS3), NS4a, NS5a, and RNA-dependent RNA polymerase (NS5b). HHpred analysis of the protein sequence found sequence similarity with hepatitis C virus envelope protein E2 (6MEHC, *e*-value = 3.1 × 10^−25^) and p7 (3ZD0, *e*-value = 0.015). According to ICTV guidelines, hepaciviruses with less than 0.25 amino acid p-distances (i.e., amino acid changes per sequence length) in a conserved region of NS3 are conspecific [58]. Since the NS3 region p-distances were less than 0.05 between MgHVs and hepacivirus F isolated from a bank vole captured from the Netherlands, the Ukrainian hepaciviruses we identified are strains of hepacivirus F.

### 3.5. Mosavirus

*Mosavirus* (mouse stool associated picornavirus) is a novel genus in the *Picornaviridae* family. There are currently only four identified mosavirus genomes, and thus the genus is not yet well defined. The first described mosavirus genome was isolated from a stool sample of North American canyon mouse (*Peromyscus crinitus*) [2], the second genome from a stool sample of a Hungarian European roller (*Coracias garrulus garrulus*) [59], the third genome from a stool sample of Himalayan marmot (*Marmota himalayana*) [60], and the fourth, partial genome from pharyngeal/anal swabs of Chinese long-tailed field mouse (*Apodemus syhaticus*) [1]. As European rollers are known to prey upon rodents, it was speculated that the mosavirus may have been derived from an infected prey [59]. The Ukrainian bank vole mosavirus genome is 9762 nt long (Figure 2g). Mosaviruses are positive-strand RNA viruses and code for a multifunctional polyprotein cleaved into four capsid proteins (VP1-VP4), three proteases (L, 2A, and 3C), and seven replication-associated proteins (2B, 2C, 3A, 3B1, 3B2, 3C, and 3D). The MgMV1 sequence coded for a protein of 2365 aa, which was a most similar to the mosavirus sequence derived from the European roller (YP009026384, 95% cover, 49.7% identity). We identified conserved domains similar to three picornavirus capsid protein domains (VP1-VP3), protease 3C, and RNA-dependent RNA polymerase 3D. Hhpred analysis of the MgMV1 found similarities to leader protease (L, 6FFA_A, *e*-value = 6.4 × 10^−14^) and VP4 (5C8C_B, *e*-value = 8.1 × 10^−46^) of foot-and-mouth disease virus and protease 2A of human poliovirus 1 (5Z3Q_D, *e*-value = 3.0 × 10^−16^). Foot-and-mouth disease virus and poliovirus are both picornaviruses. The Ukrainian bank vole mosavirus clustered with the type A mosaviruses (Figure 2h), and thus was similar to those found in the canyon mouse and European roller.

### 3.6. Viral Loads in Plasma 

**Figure 3:**
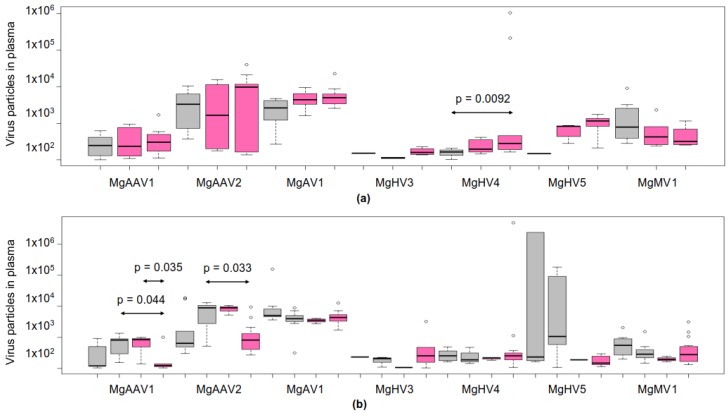
Number of plasma virus particles by qPCR in (**a**) males and (**b**) females collected from control (grey) and Chernobyl exclusion zone (pink) areas. For DNA viruses MgAAV1 and MgAAV2, the virus particle amounts were equal to 7 µL of plasma and for RNA viruses to 6 µL of plasma. The virus amounts were statistically compared pairwise using Wilcoxon rank sum test. Holm’s adjusted *p*-values are shown for groups that significantly differed from each other.

We collected plasma samples from bank voles inhabiting two control areas near Kyiv (Kyiv east: *N*_females_ = 9, *N*_males_ = 10, and Kyiv west: *N*_females_ = 10) and two sites in the Chernobyl exclusion zone (CEZ east: *N*_females_ = 3, *N*_males_ = 9 and CEZ west: *N*_females_ = 17, *N*_males_ = 10) (Figure 1b,c, and Tables S2 and S3). Virus particles were isolated from plasma, quantified by qPCR, and blotted separately for male and female bank voles according to collection site (Figure 3a,b). We did not analyze viral particle load for MgHV2, because we were not able to design specific primers for its genome. We did not find consistent statistical differences in plasma virus amounts between environmental-radiation-exposed and control populations (Tables S4 and S5), nor did we find any significant correlations between whole-body internal, external, or total absorbed dose rates (mGy/day) data derived from individual-level ***γ***-spectrometry and virus plasma amounts (Tables S2, S3 and S5, and Appendix A). However, we found some statistically significant differences in virus amounts between sampling sites: males had significantly more plasma MgHV4 virus particles in CEZ west than in control areas (Holm’s adjusted *p*-value in pairwise Wilcoxon rank sum test = 0.0092). In females, MgAAV1 amounts in plasma were significantly lower in CEZ west animals than in animals collected from CEZ east (*p* = 0.044) or from control east (*p* = 0.033). In addition, MgAAV2 plasma amounts in females were lower in the CEZ west than in control east area (*p* = 0.033).

Although female and male plasma samples were collected several months apart (July–September, 2016), the plasma virus amounts were similar between the sexes at each collection site. The only statistically significant difference in plasma viral load between females and males was that females had more MgAV1 in plasma than males in control east (*p* = 0.043). Median values for virus plasma amounts for all animals (irrespective of host collections site or sex) were MgAAV1 = 241, MgAAV2 = 1695, MgAV1 = 4182, MgHV3 = 166, MgHV4 = 205.5, MgHV5 = 282.5, and MgMV1 = 375.6 (Figure 3a,b).

### 3.7. Viral Prevalence in the Populations

**Figure 4:**
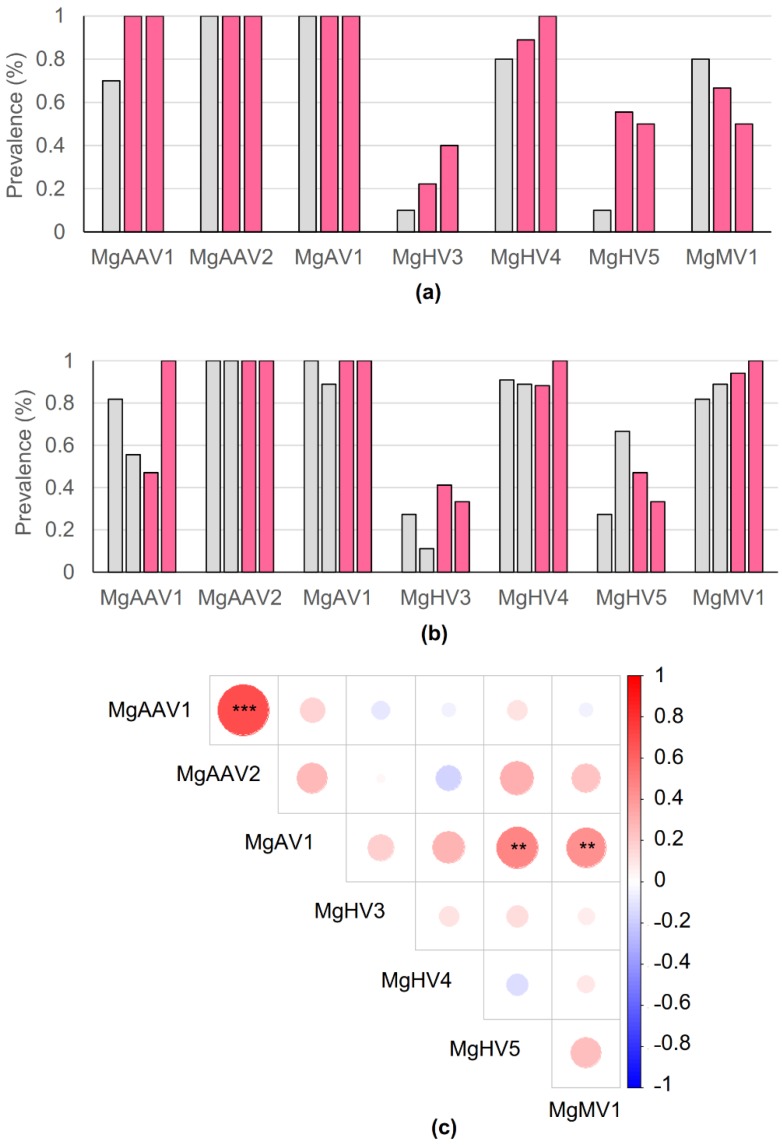
Prevalence of identified bank vole plasma viruses in (**a**) males and (**b**) females collected from control (grey) and Chernobyl exclusion zone (pink) areas. (**c**) Pairwise correlation of plasma virus particle amounts in all bank vole samples (*N* = 69) were studied by Spearman’s correlation coefficient. Positive correlations are shown in red and negative in blue. Magnitude of Holm’s adjusted *p*-values are represented by the size of the colored square. Significant values are marked with asterisks: *** *p*-value < 0.001 and ** *p*-value < 0.01.

Virus prevalences in plasma samples were analyzed separately for each sex and across populations (Figure 4a,b). The most prevalent viruses, MgAV1 and MgAAV2, were detected from almost all (i.e., 50–100%) of the bank vole samples, while the least prevalent virus, MgHV3, was detected in only 10–40% of the bank vole plasma samples. Virus prevalences did not differ significantly between samples from the Chernobyl exclusion zone and the control sites except for hepaciviruses, which were more prevalent in male (but not female) bank voles inhabiting the CEZ than in control areas. In general, virus prevalences were similar between sexes. Significant differences were found with Fisher’s exact test only between males and females for MgMV1 (*p*-value = 0.0167) and MgAAV1 (*p*-value = 0.0132). Males had MgMV1 more frequently than females and females had MgAAV1 more frequently than males. 

### 3.8. Plasma Loads of the Adeno-Associated Viruses Correlated Positively with Each Other

Because we identified two adeno-associated viruses, in which replication may depend on a helper virus [61], we studied pairwise correlations between the virus loads (Figure 4c), as we assumed that the amounts of the potential helper virus would correlate positively with the amounts of the adeno-associated viruses. There was a strong positive correlation between the amounts of the two adeno-associated viruses (Spearman correlation = 0.697, Holm’s method adjusted *p*-value < 0.0001), and also between MgAV1 and MgHV5 (correlation = 0.462, *p*-value = 0.0013) and MgMV1 (correlation = 0.428, *p*-value = 0.0047). The correlation analyses were done also separately for males and females (Figure S1A,B), and the only consistent correlation was between amounts of MgAAV1 and MgAAV2. 

### 3.9. Virus Sequences in Bank Vole Liver and Spleen Transcriptomes

**Figure 5:**
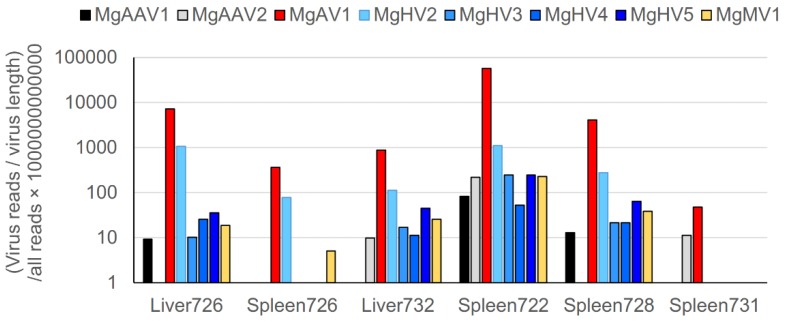
Virus read amounts in bank vole liver and spleen transcriptome samples. Properly paired virus sequences were standardized by dividing the amount of virus-specific reads with virus length and all raw reads, and multiplying by 10^12^.

We used spleen and liver RNA-sequencing data for bank voles from Ukraine [40] (NCBI SRA: SRP15797) to test whether the plasma viruses were able to produce systemic infection. Since the RNA-sequencing data were poly-A selected and the genomes of mosavirus and hepacivirus sequences did not have a clear poly-A track, our analysis might have underestimated viral load by showing fewer virus sequences than actually existed in the samples for these viruses. Spleen and liver RNAseq (transcriptome) data comprised sequence data for 40 female bank voles collected from the same locations as the samples for this study (i.e., two control locations near Kyiv, and two locations within the Chernobyl exclusion zone). Five animals had properly paired virus reads in their liver and/or spleen samples (Figure 5), all from the Kyiv east location. Notably, animal number 726 had all except MgAAV2 viruses in high amounts in its liver, and MgAV1, MgHV2, and MgMV1 in its spleen. Only MgAV1 was found in all of the five virus-positive animals, and it had the highest read amounts (on average, 6310 reads per sample). There were no properly paired virus reads in the samples from the two CEZ areas (*N* = 20) or the other control location near Kyiv (*N* = 10). 

To estimate whether viruses had caused long-term adverse effects in these five animals, we compared their body condition index to the animals without virus reads in spleen or liver [40,62]. The body condition index was calculated as the standardized residual values from a linear regression of weight against head width, where positive values reflect better body condition. We found no significant differences in the body condition index between the animals with or without virus reads (*N* = 40, Mann–Whitney: U = 61, *p* = 0.298).

To investigate the presence of the newly classified virus strains in other bank vole populations, we also searched for the virus sequences from all available wild bank vole liver and spleen transcriptomes (NBCI bioproject IDs: PRJNA429463 from England and the Netherlands, PRJNA249058 from Switzerland, and PRJNA222572 from Poland and PRJNA429463 from Sweden). We identified all of the eight virus sequences from five Swedish adult bank vole spleens (Figure S2). There were fewer aligned reads in the samples from Sweden than in those from Ukraine. In addition, hepaciviruses, not MgAV1, were the most abundant virus type in the Swedish bank voles (Figure S2).

## 4. Discussion

We identified four new bank vole virus genomes from three families, increasing the known diversity of viruses in bank voles to nearly 20 species [1,6,7,8,9,10,11,12,13,14,15,16]. The new virus genomes were not endemic to Ukraine, but were present also in Swedish bank voles, raising the possibility that these viruses are present in bank voles throughout much of their geographic range. It has been estimated that viral numbers in zoonotic reservoir species are underestimated due to inadequate sampling [63], especially in species like bank voles, which are abundant and widely distributed and may thus have many sympatric interactions that promote virus diversity [64]. Wu et al. [1] studied Chinese rodent viromes using a metagenomic analysis of pharyngeal and anal swabs, and among the rodents studied were the bank vole congeners the Grey red-backed vole (*Myodes rufocanus*) and the Northern red-backed vole (*M. rutilus*). Similar to Wu et al. [1], we found parvoviruses, mosavirus, and arterivirus from bank vole plasma, but in contrast to the study by Wu et al. [1], we also detected hepaciviruses but did not find astro-, corona-, or hantaviruses. These differences in virome composition may reflect the more restricted area of sampling and our use of plasma rather than pharyngeal and anal swabs. Thus, a greater diversity of bank vole viruses are likely to be uncovered given an increased sampling effort across the bank vole’s geographic distribution.

The two adeno-associated parvoviruses, MgAAV1 and MgAAV2, are the first known parvoviruses from bank voles. A positive correlation in MgAAV1 and MgAAV2 plasma viral load within individuals implies that their replication is regulated by a common factor, such as through the action of a (presently unknown) helper virus or some inherent feature of the host that allows both viruses to become co-abundant. Adeno-associated viruses are not known to be pathogenic, which is in line with our results, as the prevalence and plasma particle amounts of MgAAV2 were the highest among the identified viruses, yet the read amounts in the RNAseq data from liver and spleen were among the lowest, indicating that the viruses did not infect the tissues. Viral pathogenesis generally requires the virus to be able to spread in the body and multiply to large enough numbers.

The arterivirus MgAV1 had very high prevalence (90–100%) and particle amounts in plasma samples in comparison to other viruses; however, unlike the adeno-associated viruses, MgAV1 also had the highest read amounts in spleen and liver transcriptomes, indicating that it may have caused infection. Spleen and liver are lined with macrophages that ingest pathogens and activate immune defenses. Several viruses, including arteriviruses [57], infect macrophages to gain access to internal organs and to establish a persistent infection [65]. For example, porcine reproductive and respiratory syndrome virus, which is phylogenetically similar to MgAV1, causes reproductive disorders, pneumonia, and growth reduction in swine through infection of alveolar macrophages and cells of monocyte/macrophage lineage [57]. In bank voles, viral genetic material can be found from liver and spleen transcriptomes because the viruses have either (i) been transported there in blood, (ii) been engulfed by resident macrophages, or (iii) infected other resident cells. For example, bank vole hepacivirus F and some adeno-associated virus strains can infect hepatocytes [16,66]. Thus, the high amounts of virus sequence reads detected in liver and spleen transcriptome may indicate virus tropisms to macrophages, pathogenic potential, or both. Additionally, the mosavirus MgMV1 was found in bank vole blood, spleen, and liver. Tropisms of mosaviruses are not yet known, as only four mosavirus sequences have been identified thus far [1,2,59,60]. However, our results show that the MgMV1 mosavirus is not food-associated and that it may cause active viremia. 

The fact that fewer animals (12.5%) had detectable amounts of virus in their liver and spleens (transcriptome analysis) than in plasma (where virus particle prevalences were >50% according to qPCR analysis) indicates that these apparently common (at least in the Ukrainian bank voles) viruses rarely become systemic. Interestingly, the animals that had viral genetic material in their liver or spleen were apparently infected by most of the viruses, which may indicate generally poor health or a compromised immune system, which aided virus replication and infection. However, the five Ukrainian bank voles from the transcriptome analysis that had viruses in spleen and/or liver did not have lower body condition index compared to the animals without viruses in these tissues, indicating that these animals had not suffered from poor body condition for extended period of time. Thus, the pathogenic potential of the identified viruses remains unresolved. 

Counter to our expectations, we found no significant differences in virus characteristics between bank voles from high environmental radiation and control areas. Significant differences in plasma viral load and prevalence among collection sites could indicate that viral dynamics are more dependent on the local ecology and demography of the host population rather than radionuclide contamination.

## Figures and Tables

**Table 1:** Remapping reads to the identified virus sequences. Only properly paired reads are shown.

Virus	Genbank ID	Length (nt)	Reads Kyiv West	Reads Kyiv East	Reads CEZ	All Reads	Average Coverage
MgAAV1	MN242366	4927	1626	131	0	1757	35.66
MgAAV2	MN242367	4517	0	1108	102	1210	26.79
MgAV1	MN242368	15,397	0	43,566	0	43,566	282.95
MgHV2	MN242369	8900	272,994	0	0	272,994	3067.35
MgHV3	MN242370	8838	143,029	414,151	7,165,765	7,722,945	87,383.40
MgHV4	MN242371	8838	961	1033	2,312,332	2,314,326	26,186.08
MgHV5	MN242372	8828	0	196,742	0	196,742	2228.61
MgMV1	MN242373	9762	4363	5379	248	9990	102.34

## Supplementary Materials

The following are available online at https://www.mdpi.com/1999-4915/12/1/44/s1: Figure S1: Pairwise correlation of plasma virus particle amounts in males and females; Figure S2: Virus amounts in Swedish bank vole spleen transcriptome samples; Table S1: Bank vole females collected on October 2016 used for NGS, Table S2: Female bank vole samples used in virus qPCR analysis; Table S3: Male bank vole samples used in virus qPCR analysis; Table S4: Pairwise comparisons of virus amounts between females of Chernobyl exclusion zone and control populations; Table S5: Pairwise comparisons between absorbed dose rates and virus amounts; Table S6: Virus-specific qPCR primers used in this study.

## Appendix A

### Appendix A.1. DOSIMETRY

#### Appendix A.1.1. External Absorbed Dose Rate Estimations

We measured ambient radiation levels with the use of a hand-held Geiger counter (Gamma-Scout, GmbH & Co., Germany), placed at 1 cm above the ground.We took at least 9 measurements at each trapping location and used the mean value for each location in the analysis. The ambient dose rate measurements are thought to give a reasonable approximation of the external absorbed dose rates for bank, however such approximations could underestimate external radiation dose rates in case of other species. With the use of implanted thermo-luminescent dosimeters for direct measurement of the external absorbed doses (as well as absorbed dose rate) for bank voles inhabiting the Chernobyl Exclusion Zone we provide empirical support for such estimations (Lavrinienko et al., submitted). Thus, the external absorbed dose rates were (mean ± SD): 0.00495 ± 0.00175 mGy/day for the bank voles captured from the Kiev east and west areas, and 0.44814 ± 0.26044 mGy/day for voles inhabiting the Chernobyl east and west areas.

#### Appendix A.1.2. Internal Absorbed Dose Rate Estimations

To estimate internal radiation dose rate due to the radiocaesium contamination, ^137^Cs activity was measured in each bank vole individual with the SAM 940 radionuclide identifier system (Berkeley Nucleonics Corporation, San Rafael, CA, USA) equipped with a 3″ × 3″ NaI detector. The detector was enclosed in 10 cm thick lead shielding to reduce the noise from background radioactivity. The system was calibrated with reference standard sources. With corrections for laboratory background, the ^137^Cs activity of whole bodies was evaluated from the obtained spectra in the energies range 619–707 keV (with caesium photopeak at 662 keV) with the use of the phantom with known activity and geometry similar to the samples. Given the additional time animals spent from capture to the actual processing, the initial ^137^Cs activity (at the trapping timepoint) was calculated with the model *A* = *be^λx^*, where: *A* is calculated initial caesium activity; *b* is activity that was measured in animal bodies; *x* is time in hours (after trapping and before sacrificing); *e* is 2.72; *λ* is elimination constant.

According to for each measurement the critical detectable level (decision threshold) was found as *Lc* = *k*[*Rb*/*Tb*(1 + *Tb*/*Ts*]^1/2^, where: *Lc* is critical level; *k* is 1.65 (coefficient, which determine 0.05 probability of type I error or false positive); *Rb* is counting rate of background, *Tb* is time of background measurement, *Ts* is time of sample measurement. The caesium activities above the critical level (decision threshold) were used for internal dose rate estimation; otherwise, the ^137^Cs activity was treated as a zero.

An individual internal absorbed dose from incorporated caesium-137 acquired during one day was calculated as:

$$D_{\mathrm{int}}=A*C_{u}*(\sum_{i}\phi_{i}*E_{i}*f_{i})$$

where: *D*_int_ is the absorbed dose rate due to internal exposure from ^137^Cs (mGy/day); *A* is the activity of ^137^Cs incorporated in the animal body (Bq/kg); *C_u_* is the unit conversion coefficient; *ϕ_i_* is the absorbed fractions for electron, positron or photon of the specific energy line *E_i_* self-absorption in tissue for the ^137^Cs source which is uniformly distributed throughout homogeneous sphere of mass 20 g of unit density and tissue-equivalent composition (Stabin et al., 2000); *f_i_* is the intensity (or emission frequency) of the specific energy line *E_i_* (MeV) emitted per decay of ^137^Cs and it daughter radionuclide ^137^mBa (ICRP Publication 38, 1983), the sum is taken over all electron, positron and photon energies *E_i_* of ^137^Cs spectrum (see also Chesser et al., 2000).

Thus, the internal absorbed dose rates from ^137^Cs were: ranging from 0.0 to 0.00149 mGy/day in animals captured from Kiev east and west areas, and in range of 0.00328-1.74251 mGy/day from Chernobyl east and west areas.Despite of considerable contribution of ^90^Sr to the total internal absorbed dose, which is considered to be similar to that from ^137^Cs (Beresford et al., 2018), in our study we neglected it because of characteristics of this beta-emitting radionuclide (tissue-specific strontium distribution: ^90^Sr deposits mainly in bones).

#### Appendix A.1.3. Total Absorbed Dose Rate Estimations

The total absorbed dose rates were estimated as the sum of the internal and external absorbed dose rates estimates for each animal.
