# Supplementary material for: Infection Load and Prevalence of Novel Viruses Identified from the Bank Vole Do Not Associate with Exposure to Environmental Radioactivity
Source: Viruses. 2019 Dec 30;12(1):44. doi: 10.3390/v12010044 (PMC7019477; doi:10.3390/v12010044)
Supplement: Supplementary file 1 [file viruses-12-00044-s001.pdf]

Supplementary material

(a)

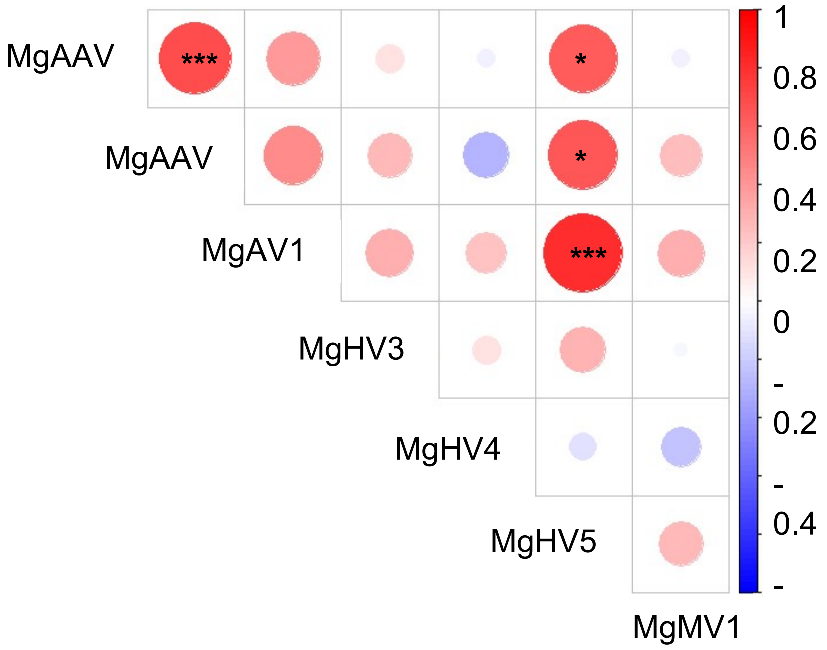

(b)

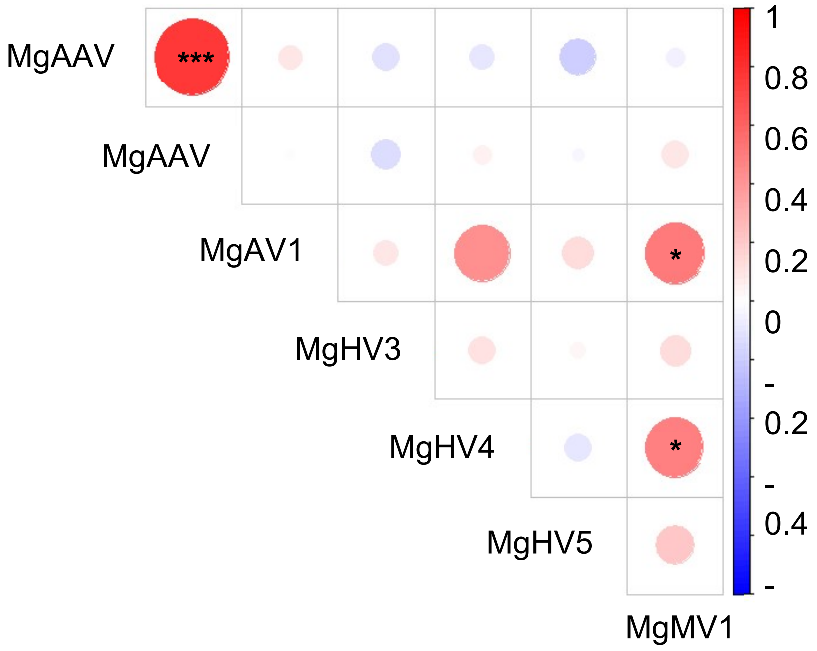

Figure S1. Pairwise correlation of plasma virus particle amounts in males and females. Plasma virus particle amounts in A) male (N=29) and B) female (N=40) bank voles were studied by Spearman's correlation coefficient. Positive correlations are shown in red and negative in blue. Magnitude of Holm's adjusted p-values are represented by the size of the

coloured square. Significant values are marked with asterisk (*** = p-value < 0.001, ** = p-value < 0.01, * = p-value < 0.05).

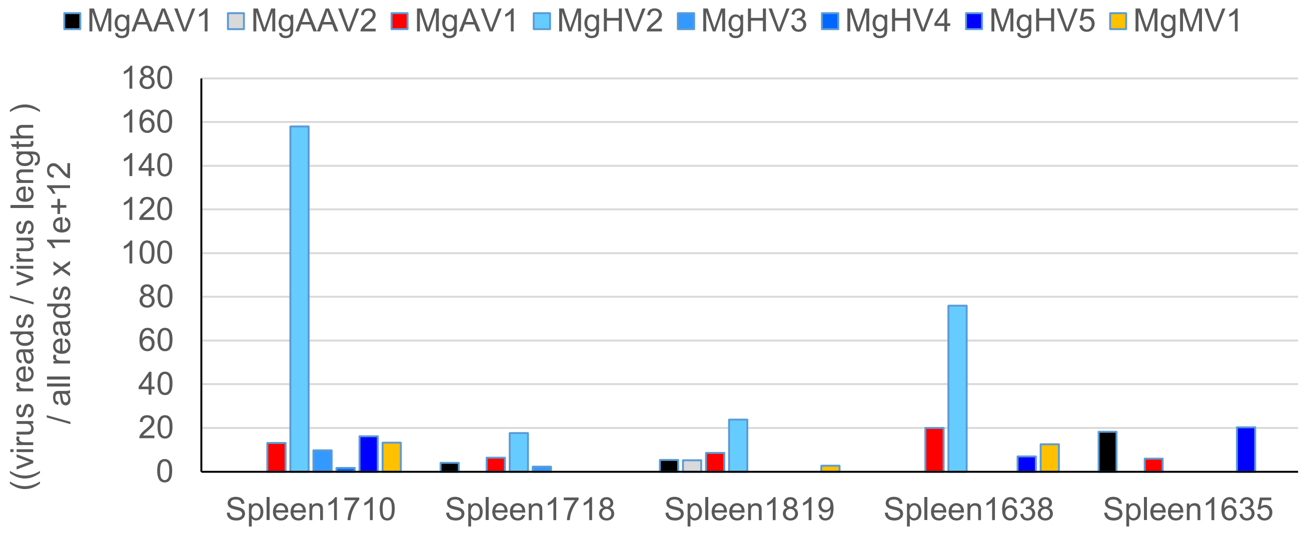

Figure S2. Virus amounts in Swedish bank vole spleen transcriptome samples (NBCI bioproject ID: PRJNA429463). Properly paired virus sequences were standardised by dividing the virus-specific read amount with virus length and all raw reads and multiplying by 100 000 000 000.

Table S1. Bank vole females collected on October 2016 used for NGS.

Animal no.	Collection place	Collection date	Body mass, g	Internal dose rate, mGy/day (^{137}Cs only)	External dose rate, mGy/day	Total dose rate, mGy/day	Pool
55	Kyiv west	04.10.2016	18.03	0.00000	0.00362	0.00362	CTRL1
60	Kyiv west	04.10.2016	18.61	0.00000	0.00362	0.00362	CTRL1
62	Kyiv west	04.10.2016	16.49	0.00000	0.00362	0.00362	CTRL1
63	Kyiv west	04.10.2016	16.23	0.00000	0.00362	0.00362	CTRL1
67	Kyiv west	04.10.2016	17.71	0.00000	0.00362	0.00362	CTRL1
70	Kyiv west	04.10.2016	16.86	0.00000	0.00362	0.00362	CTRL1
82	Kyiv west	04.10.2016	19.75	0.00000	0.00362	0.00362	CTRL1
94	Kyiv west	04.10.2016	16.95	0.00000	0.00362	0.00362	CTRL1
84	Kyiv west	04.10.2016	16.58	0.00059	0.00362	0.00421	CTRL1
2	Kyiv east	05.10.2016	17.22	0.00000	0.00720	0.00720	CTRL2
10	Kyiv east	05.10.2016	23.34	0.00000	0.00720	0.00720	CTRL2
33	Kyiv east	05.10.2016	16.89	0.00000	0.00720	0.00720	CTRL2
44	Kyiv east	05.10.2016	16.64	0.00000	0.00720	0.00720	CTRL2
7	Kyiv east	05.10.2016	19.82	0.00062	0.00720	0.00782	CTRL2
12	Kyiv east	05.10.2016	20.76	0.00063	0.00720	0.00783	CTRL2
22	Kyiv east	05.10.2016	16.87	0.00073	0.00720	0.00793	CTRL2
6	Kyiv east	05.10.2016	21.61	0.00084	0.00720	0.00804	CTRL2
72	CEZ west	09.10.2016	15.60	0.00669	0.26400	0.27069	CEZ
63	CEZ west	09.10.2016	14.92	0.00830	0.26400	0.27230	CEZ
71	CEZ west	09.10.2016	15.90	0.01166	0.26400	0.27566	CEZ
35	CEZ west	09.10.2016	15.08	0.01393	0.26400	0.27793	CEZ
37	CEZ west	09.10.2016	18.11	0.02206	0.26400	0.28606	CEZ
38	CEZ west	09.10.2016	18.75	0.02938	0.26400	0.29338	CEZ
74	CEZ west	09.10.2016	16.21	0.03120	0.26400	0.29520	CEZ
44	CEZ west	09.10.2016	16.44	0.03191	0.26400	0.29591	CEZ
78	CEZ west	09.10.2016	15.06	0.00969	0.40800	0.41769	CEZ
53	CEZ west	09.10.2016	14.83	0.00605	0.44616	0.45221	CEZ

Table S2. Female bank vole samples used in virus qPCR analysis.

Animal no.	Collection place	Collection date	Body mass,g	Internal dose rate, mGy/day (^{137}Cs only)	External dose rate, mGy/day	Total dose rate, mGy/day
66	Kyiv west	05.10.2016	16.69	0.00000	0.00362	0.00362
68	Kyiv west	05.10.2016	14.50	0.00000	0.00362	0.00362
76	Kyiv west	05.10.2016	15.30	0.00000	0.00362	0.00362
85	Kyiv west	05.10.2016	15.88	0.00000	0.00362	0.00362
90	Kyiv west	05.10.2016	15.66	0.00000	0.00362	0.00362
107	Kyiv west	05.10.2016	15.64	0.00000	0.00362	0.00362
105	Kyiv west	05.10.2016	23.57	0.00010	0.00362	0.00372
100	Kyiv west	05.10.2016	14.57	0.00010	0.00362	0.00372
79	Kyiv west	05.10.2016	24.13	0.00030	0.00362	0.00393
75	Kyiv west	05.10.2016	15.62	0.00031	0.00362	0.00393
14	Kyiv east	05.10.2016	15.49	0.00000	0.00720	0.00720
20	Kyiv east	05.10.2016	23.06	0.00000	0.00720	0.00720
30	Kyiv east	05.10.2016	14.01	0.00000	0.00720	0.00720
34	Kyiv east	05.10.2016	18.19	0.00000	0.00720	0.00720
37	Kyiv east	05.10.2016	18.42	0.00000	0.00720	0.00720
39	Kyiv east	05.10.2016	14.91	0.00000	0.00720	0.00720
40	Kyiv east	05.10.2016	17.40	0.00000	0.00720	0.00720
42	Kyiv east	05.10.2016	13.59	0.00000	0.00720	0.00720
21	Kyiv east	05.10.2016	24.09	0.00023	0.00720	0.00743
47	CEZ west	09.10.2016	11.15	0.00328	0.26400	0.26728
66	CEZ west	09.10.2016	15.66	0.00408	0.26400	0.26808
68	CEZ west	09.10.2016	16.00	0.02358	0.26400	0.28758
33	CEZ west	09.10.2016	16.58	0.04972	0.26400	0.31372
82	CEZ west	09.10.2016	12.74	0.00432	0.36000	0.36432
21	CEZ west	09.10.2016	16.72	0.02534	0.36000	0.38534
22	CEZ west	09.10.2016	13.00	0.03234	0.36000	0.39234
50	CEZ west	09.10.2016	14.13	0.04070	0.36000	0.40070
30	CEZ west	09.10.2016	21.46	0.09021	0.36000	0.45021
81	CEZ west	09.10.2016	12.27	0.01361	0.39168	0.40529
16	CEZ west	09.10.2016	13.66	0.14603	0.39168	0.53771
55	CEZ west	09.10.2016	14.18	0.00888	0.40800	0.41688
34	CEZ west	09.10.2016	18.85	0.06906	0.40800	0.47706
26	CEZ west	09.10.2016	16.89	0.01730	0.44616	0.46346
79	CEZ west	09.10.2016	19.17	0.01765	0.44616	0.46381
56	CEZ west	09.10.2016	13.92	0.01804	0.44616	0.46420
57	CEZ west	09.10.2016	13.78	0.03074	0.45600	0.48674
59	CEZ east	09.10.2016	21.59	0.53218	1.10226	1.63444
9	CEZ east	09.10.2016	17.29	1.13526	1.10226	2.23752

62	CEZ east	09.10.2016	21.05	1.74251	1.10226	2.84477

Table S3. Male bank vole samples used in virus qPCR analysis.

Animal no.	Collection place	Collection date	Body mass,g	Internal dose rate, mGy/day (^{137}Cs only)	External dose rate, mGy/day	Total dose rate, mGy/day
933	CEZ east	24.07.2016	17.77	0.04625	0.25542	0.30167
888	CEZ east	24.07.2016	19.86	0.11447	0.27132	0.38579
923	CEZ east	24.07.2016	20.07	0.01931	0.28707	0.30638
937	CEZ east	24.07.2016	15.67	0.03630	0.44112	0.47742
938	CEZ east	24.07.2016	17.49	0.12961	0.44112	0.57073
924	CEZ east	24.07.2016	17.02	0.21886	0.44112	0.65998
885	CEZ east	24.07.2016	20.65	0.24024	0.84129	1.08153
936	CEZ east	24.07.2016	24.86	0.44026	0.84129	1.28155
886	CEZ east	24.07.2016	15.76	0.63949	1.49256	2.13205
908	CEZ west	24.07.2016	18.00	0.03920	0.36000	0.39920
907	CEZ west	24.07.2016	22.61	0.01515	0.39168	0.40683
896	CEZ west	24.07.2016	21.31	0.02368	0.39168	0.41536
904	CEZ west	24.07.2016	26.20	0.02464	0.40800	0.43264
902	CEZ west	24.07.2016	17.51	0.02848	0.40800	0.43648
898	CEZ west	24.07.2016	19.52	0.03770	0.40800	0.44570
903	CEZ west	24.07.2016	23.30	0.10446	0.40800	0.51246
900	CEZ west	24.07.2016	20.02	0.02809	0.44616	0.47425
899	CEZ west	24.07.2016	17.91	0.03769	0.44616	0.48385
906	CEZ west	24.07.2016	23.35	0.01842	0.45600	0.47442
79	Kyiv west	18.07.2016	15.40	0.00000	0.00362	0.00362
80	Kyiv west	18.07.2016	16.50	0.00000	0.00362	0.00362
84	Kyiv west	18.07.2016	20.70	0.00000	0.00362	0.00362
91	Kyiv west	18.07.2016	17.30	0.00000	0.00362	0.00362
100	Kyiv west	18.07.2016	17.20	0.00000	0.00362	0.00362
102	Kyiv west	18.07.2016	17.80	0.00000	0.00362	0.00362
106	Kyiv west	18.07.2016	18.90	0.00000	0.00362	0.00362
109	Kyiv west	18.07.2016	15.60	0.00000	0.00362	0.00362
121	Kyiv west	18.07.2016	16.10	0.00000	0.00362	0.00362
97	Kyiv west	18.07.2016	15.50	0.00149	0.00362	0.00511

Table S4. Pairwise comparisons of virus amounts between males from Chernobyl Exclusion Zone (CEZ east and west) and control populations (Kyiv west) using Wilcoxon rank sum test with Holm adjusted p-value.

**Males**		
**MgAAV1**	CEZ east	Kyiv west
Kyiv west	1	NA
CEZ west	1	1
**MgAAV2**		
Kyiv west	1	NA
CEZ west	1	1
**MgAV1**		
Kyiv west	0.11	NA
CEZ west	0.73	0.07
**MgHV2**		
Kyiv west	1	NA
CEZ west	0.4	1
**MgHV3**		
Kyiv west	1	NA
CEZ west	1	1
**MgHV4**		
Kyiv west	0.166	NA
CEZ west	0.237	0.0092
**MgMV1**		
Kyiv west	0.56	NA
CEZ west	0.93	0.51

Table S4. Pairwise comparisons of virus amounts between females of Chernobyl Exclusion Zone (CEZ west) and control populations (Kyiv east and west) using Wilcoxon rank sum test with Holm adjusted p-value.

**MgAAV1**	CEZ east	Kyiv east	Kyiv west
Kyiv east	0.75	NA	NA
Kyiv west	1	0.67	NA
CEZ west	0.67	1	0.033
**MgAAV2**			
Kyiv east	0.63	NA	NA
Kyiv west	1	0.32	NA
CEZ west	0.035	1	0.0044
**MgAV1**			
Kyiv east	0.15	NA	NA
Kyiv west	0.92	0.31	NA
CEZ west	0.92	0.56	0.92
**MgHV3**			
Kyiv east	1	NA	NA
Kyiv west	1	1	NA
CEZ west	1	1	1
**MgHV4**			
Kyiv east	1	NA	NA
Kyiv west	1	1	NA
CEZ west	1	0.64	1
**MgHV5**			
Kyiv east	1	NA	NA
Kyiv west	1	1	NA
CEZ west	1	1	1
**MgMV1**			
Kyiv east	0.29	NA	NA
Kyiv west	1	1	NA
CEZ west	1	1	1

Table S5. Pairwise comparisons between absorbed dose rates and virus amounts. Whole-body internal (INT), external (EXT), or total (TOT) absorbed dose rates (mGy/day) and virus amounts in all qPCR samples (N=43) were compared using Spearman correlation (corr) with Holm adjusted p-value (p-value).

	**MgAAV1**	**MgAAV2**	**MgAV1**	**MgHV3**	**MgHV4**	**MgHV5**	**MgMV1**
**INT**	-0.04	-0.1	0.3	0.38	0.13	0.15	0.15
**p-value**	1	1	1	0.43	1	1	1
**EXT**	-0.22	-0.19	0.33	0.28	-0.019	0.13	0.17
**p-value**	1	1	1	1	1	1	1
**TOT**	-0.2	-0.18	0.31	0.29	0.061	0.0613	0.18
**p-value**	1	1	1	1	1	1	1

Table S6. Virus-specific qPCR primers used in this study.

**Virus**	**Forward primer (5')**	**Reverse primer (5')**
MgAAV1	CATAAAAGGAAGCGCGAGAC	GCAGATTCTTCCTCCGTCAG
MgAAV2	GAGCGTCTGCAGGGAGATAC	GTCTCGTCTCCTTCGACCAG
MgAV1	TTCGGCCAGTATGTGGTGTA	AAATTCAGCCTTCTGCCTCA
MgHV3	ACCATCTTGGGTATTGGGTCTGTAC	GAACTCCCCTTCGTCTGAGAGG
MgHV4	GCCGCTAGGGTGGTGGAGTAC	GGACGCTGCCCAGTCCG
MgHV5	GTTTATTGACCCATTCTTCCCCTGT	GCAGGATTCAAGCCCTCCTT
MgMV1	ACAGAAATGTACGCCGCTGA	TGTCACGCCATCACCATTCA
